# Rheological, Technological, and Nutritional Profile of Sustainable Crops: Bread Wheat Evolutionary Populations

**DOI:** 10.3390/foods14223821

**Published:** 2025-11-07

**Authors:** Chiara Natale, Elena Galassi, Francesca Nocente, Federica Taddei, Silvia Folloni, Giovanna Visioli, Salvatore Ceccarelli, Gianni Galaverna, Laura Gazza

**Affiliations:** 1 CREA Research Centre for Engineering and Agro-Food Processing, Via Manziana 30, 00189 Rome, Italy; chiara.natale@crea.gov.it (C.N.); elena.galassi@crea.gov.it (E.G.); francesca.nocente@crea.gov.it (F.N.); federica.taddei@crea.gov.it (F.T.); 2 Open Fields S.r.L., Colorno, 43126 Parma, Italy; s.folloni@openfields.it; 3 Department of Chemistry, Life Sciences and Environmental Sustainability, University of Parma, Parco Area delle Scienze 11/a, 43121 Parma, Italy; giovanna.visioli@unipr.it; 4 Independent Researcher, 63100 Ascoli Piceno, Italy; ceccarelli.salvatore83@gmail.com; 5 Department of Food and Drug, University of Parma, Parco Area delle Scienze 27/a, 43121 Parma, Italy; gianni.galaverna@unipr.it

**Keywords:** climate-smart food, agroecology, seed mixtures, agrifood biodiversity

## Abstract

The present research aimed to design innovative wheat cultivation systems that are less resource-intensive, promote biodiversity, and show greater resilience to both biotic and abiotic stress. It was focused on the cultivation and characterization of two evolutionary populations (EPs) of common wheat, namely EP_Floriddia and EP_Li Rosi, grown in Italy, over two growing seasons. The EPs were cultivated in organic management under legume or wheat precessions. Physico-chemical analyses included thousand kernel weight (TKW), test weight (TW), and ash content. Location and genotype mostly influenced TKW; TW, instead, was affected only by year. Wholemeal flour from each sample was assessed for protein content (PC), total starch (TS), total antioxidant capacity (TAC), and total dietary fiber (TDF). Protein content was higher on leguminous precessions than on wheat; the opposite behavior was observed for TS. The growing season predominantly impacted on TAC and TDF values. Technological and rheological parameters such as alveograph W and P/L value, SDS sedimentation test, farinograph quality, gluten index, and falling number revealed EP_Li Rosi as the best for baking aptitude, although both EPs were characterized by weak gluten. These findings support the use of EPs under legume precession as an agroecological approach to pursue agrifood biodiversity, quality, and sustainability.

## 1. Introduction

Among cereals worldwide, in 2023, wheat was the third most produced, at 798.98 Mt, and the first for area harvested, with 220.41 Mha [1]. Intensive agricultural systems based on optimizing the productivity of monocultures are now widely criticized for their negative environmental impacts, as they degrade soil, reduce agro-food biodiversity, and rely heavily on fossil fuels and chemical inputs.

The vulnerability of crops is a huge consequence of the loss of agrobiodiversity, because their genetic uniformity makes them unable to respond to both short- and long-term climate changes [2] and promotes the rapid emergence of pesticide-resistant variants [3]. Agrobiodiversity is a key to food security, making production systems more resilient to both biotic and abiotic stresses [4]. Highly diversified cropping systems based on agroecological principles have been shown to have potential benefits in terms of biodiversity conservation and ecological sustainability [5]. Through the application of ecological principles to agriculture, ensuring the regenerative use of natural resources and ecosystem services, agroecology could contribute to transforming agrifood systems.

In this context, the cultivation of cereal evolutionary populations (EPs), mixtures of seeds obtained by crossing different varieties of the same species, relying on natural selection and evolution driven by the environment [6], is establishing itself as an agroecological approach to counteract the loss of biodiversity and the negative effects of climate change. Indeed, thanks to their high genetic diversity, composed of thousands of genetically distinct seeds, wheat EPs are capable of dynamic adaptation to the specific environmental conditions in which they are cultivated. Over successive generations, EPs undergo natural selection processes, gradually evolving in response to local climatic factors, soil characteristics, and agronomic practices. This adaptive capacity allows EPs to maintain stable yields across growing seasons, even under complex and variable stress conditions, including both biotic stresses (such as diseases, pests, and weeds) and abiotic stresses, such as drought, extreme temperatures, and salinity [6]. As better-adapted genotypes within the EPs increase in frequency over time, the reliance on external chemical inputs diminishes. This results in lower production costs, and makes EPs particularly well-suited for low-input, organic, and regenerative farming systems [7]. In 2022, the new EU organic regulation [8] came into force, establishing rules for certified organic production at the European Union level. This regulation introduced new options for reproductive plant material available to organic farmers, including evolutionary populations classified under the category of organic heterogeneous material (OHM) [9]. Bread wheat (*Triticum aestivum* L.) evolutionary populations were introduced to Italy in 2010 by the International Center for Agricultural Research in the Dry Areas (ICARDA). The two EPs studied in the present research evolved in two Italian regions (Sicily and Tuscany), characterized by two completely different pedoclimatic conditions, with Sicily being systematically drier and warmer than Tuscany [10]. After eleven years of evolution, the two bread wheat populations grown in Sicily or in Tuscany have been named Furat Li Rosi (hereafter EP_Li Rosi) and Furat Floriddia (hereafter EP_Floriddia), respectively.

EP_Floriddia and EP_Li Rosi have been studied within the UE PRIMA program project “Innovative agroecological approaches to achieve resilience to climate CHANGE in Mediterranean countries—(CHANGE-UP)”. The project, involving five countries from the Mediterranean basin, aimed to re-design innovative, less resource-demanding, and biodiversity-based cereal farming systems, more resilient to environmental constraints and climate unpredictability, by the introduction of perennial wheat lines, whose technological and nutritional traits are reported in [11], and of cereal EPs in crop rotation with legumes.

Currently, the driving forces and emerging trends within the food industry are undergoing continuous transformation. Consumers increasingly prefer producers who integrate environmental considerations and food system sustainability into food development [12]. In this study, the nutritional and technological performance of the bread wheat varieties EP_Floriddia and EP_Li Rosi, cultivated in organic management over two years in two different Italian regions, in crop rotation with legumes (chickpea, clover, and pea) and wheat, was assessed and compared with a commercial bread wheat variety. The overall objective was to investigate and valorize this novel raw material not only with the aim of enhancing agro-food biodiversity but also to explore wheat evolutionary populations for future application within the new oriented food system, aspiring to meet the rising demands of sustainability from producers and consumers.

## 2. Materials and Methods

### 2.1. Plant Materials

Two bread wheat evolutionary populations, EP_Floriddia and EP_Li Rosi, hereafter named EP_Floriddia and EP_Li Rosi, and the bread wheat variety ‘Monnalisa’, were cultivated during two growing seasons (2022 and 2023) in two Italian locations: the first in the experimental fields of CREA—Research Center for Engineering and Agri-Food Processing (CREA-IT), located in Montelibretti, (42°13′ N; 12°64′ E, 20 m asl) and the second at Stuard experimental farm located in Parma (44° 48′ N; 10°16′ E, 10 m asl). At Montelibretti, the two legumes used for precession were chickpea and clover. At Stuard experimental farm, cereals were grown after pea or wheat as preceding crops. The Monnalisa variety was chosen as the control because it is widely grown in the central-northern regions, including in organic farming, and is valued by the milling industry.

Cultivation was carried out without the use of fertilizers, herbicides, and pesticides and without irrigation at any stage. Grain samples were evaluated for physico-chemical (thousand kernel weight, test weight, and ash content), nutritional (protein content, total starch, total dietary fiber, total antioxidant capacity), rheological, and technological (alveographic and farinograph parameters, SDS sedimentation test, gluten index and falling number) characteristics. All analyses were performed in triplicate, and the data were expressed as dry basis.

### 2.2. Physico-Chemical Characterization

Thousand kernel weight (TKW) and test weight (TW) were evaluated by the ISO 520:2010 [13] and ISO 7971-1:2009 [14] methods, respectively. All samples were milled to wholemeal flour using a laboratory mill (Cyclotec, Tecator, Hoganas, Sweden) with 0.5 or 1.0 mm (for total antioxidant capacity) sieves, and kept at 4 °C until their use. Moisture content was measured using the thermobalance (Sartorius MA 40, Göttingen, Germany) at 120 °C just before the chemical analyses. Ash content was determined according to the approved method AACC 08-01.01 [15].

### 2.3. Nutritional Characterization

Protein content (PC) was determined according to the Dumas method AACC n. 46-30 [16]. Total starch (TS) was measured using the enzymatic method by a Megazyme (Bray, Ireland) kit K-TSTA according to AOAC methods 996.11 [17]. Total dietary fiber (TDF) content was determined using an enzymatic kit for fiber determination (Bioquant, Merck, Darmstadt, Germany) according to the Official Method 991.42 [18]. Total antioxidant capacity (TAC) was determined according to [19].

### 2.4. Technological and Rheological Characterization

Flours, obtained by a CD1 laboratory mill (Chopin, Villeneuve La Garenne, France), were analyzed by a Chopin Alveograph according to the manufacturer’s instructions under conditions as described by the standard AACC method 54-30.02 [20]. The SDS sedimentation test was performed according to the standard method AACC 56-70.01 [21]. Gluten index (GI) determination was conducted by the Glutomatic 2200 (Perten Instruments, Segeltorp, Sweden) according to the AACC method 38-12 [22]. The farinographic test was conducted using a Brabender Farinograph (Duisburg, Germany) following ISO 5530-1:2013 [23]. The AACC 56-81B method [24] was used for the determination of the falling number (FN) using the Perten 1500 system (Perten Instruments). Storage proteins were extracted following Osborne sequential extraction and fractionated in redox conditions by 10% *w*/*v* SDS-PAGE, as described by [25]. Three bread wheat cultivars of known allelic composition at *Glu A1*, *Glu B1*, and *Glu D1* loci were used as controls.

### 2.5. Statistical Analysis

Data were analyzed with an analysis of variance for unbalanced data set by GenStat 24th edition [26]. The comparisons between entry means were performed using Fischer’s LSD test with a significance level of 5% as available in the GenStat ANOVA for unbalanced data sets.

## 3. Results and Discussion

Given the objective of this work, aiming to contrast the unpredictability of climate change using innovative cultivation systems, the collection of the meteorological data over the two growing seasons was considered to be of fundamental importance to fully understand the performances of EPs in relation to climate variations. Total monthly rainfall (mm) and monthly mean temperature (°C) were collected from January 2022 to July 2023. No differences in the mean temperature were observed in both locations between the two growing seasons (15.7 °C and 15.1 °C in 2022 and 2023, respectively), even if 1.5 °C of difference was registered in the average T max in the two years, the first being the hottest. Conversely, in both locations, over the 2022 growing season, the total amount of rainfall was absolutely lower (72.7 mm on average) than in the 2023 season, when the mean value was 460.7 mm. Notably, besides the amount of rainfall, the period when the rain occurs can determine effects on agronomic performance and consequently on grain quality.

### 3.1. Physico-Chemical Characterization

Thousand kernel weight (TKW) and test weight (TW) are fundamental merceological parameters, as they represent the kernel shape, size, and degree of filling, and therefore the flour yield [27]. TKW results were significantly influenced by location, genotype (entry), and by the entry x precession interaction (Table 1). Indeed, TKW was significantly higher at Montelibretti than at Parma (44.62 vs. 42.01 g, respectively), and EP_Floriddia had the highest TKW (46.56 g), mainly in chickpea precession (Table 1). The significant differences observed between the locations may be attributed to the effects of the different precessions; indeed, at Montelibretti, the two preceding legume crops may have led to an increased amount of available nitrogen, resulting in higher TKW values, whereas at Parma the wheat precession was likely responsible for the lower TKW. There was a significant difference between years for TW, which was higher in 2022 than in 2023 (Table 1). The TW values after chickpea and peas were ≥78.0 kg/hL (Table 1), ascribable to the No.2 UNI quality class (TW ≥ 78 kg/hL) of the UNI 10709:1998 [28]. The significantly higher TW value observed in 2022 with respect to the 2023 growing season may be explained by the greater amount of rainfall that occurred at the seed maturity stage. Excess rainfall during this period may have caused early sprouting and swelling, ultimately leading to a lower TW value, because the larger kernels weigh the same but take up more space [29].

Ash content was significantly influenced by preceding crops, locations, and years (Table 1). The highest values were always found in EP_Floriddia regardless of precession, location, and year of cultivation; the highest ash percentage was found after chickpea precession, not significantly different from pea and clover but significantly higher than when the preceding crop was wheat (Table 1), particularly at Montelibretti. The lower TW in 2023 likely also accounted for the significantly higher ash content observed, because of the low ratio of endosperm/outmost kernel layers. Moreover, the different soil composition and preceding crops in the two locations might have contributed to the significant differences in this parameter [30].

### 3.2. Nutritional Characterization

**Figure 1:**
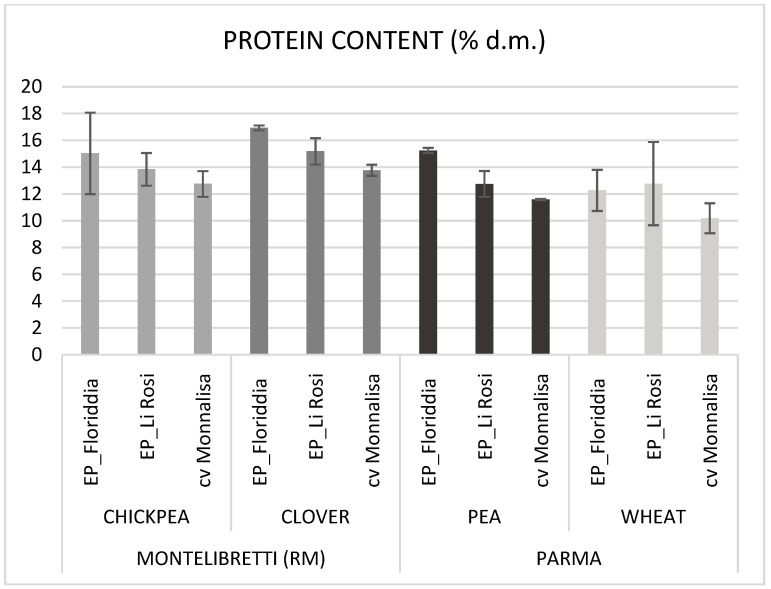
Mean protein content of EP_Floriddia, EP-Li Rosi, and bread wheat cv Monnalisa over 2022 and 2023 growing seasons, in Montelibretti (RM) and Parma, after different preceding crops.

The EPs had a significantly higher PC than cv Monnalisa (Table 2, Figure 1), with the EP_Floriddia being significantly higher (14.9%) than the EP_LI Rosi (13.6%). PC was also significantly influenced by year, location, and precession (Table 2). The preceding crop had a large effect on protein content and, as expected, the legumes determined on average a 20% increase in protein content when compared to nitrogen-depleting wheat. Among the legumes, the largest increase (up to 30%) was observed after clover. This observation confirms the well-known benefit of legumes in cropping systems, as they contribute atmospheric nitrogen through biological N_2_ fixation and recycle nitrogen-rich residues, a fundamental process for maintaining soil fertility, particularly in organic farming systems [31]. No significant differences were found between entries in TS, which, on the other hand, was significantly affected by year, location, and preceding crop. Wheat as a previous crop resulted in higher TS content than legumes, although not significantly different from pea, as a consequence of the lower protein content in kernels following a wheat precession (Table 2). Similarly, total dietary fiber (TDF) was not influenced by the entry, whereas preceding crop, year, and location affected it significantly. The highest TDF values were found in Parma in 2023. This is in agreement with the reduced TW observed in 2023, likely due to the more severe drought conditions in 2022, since small kernels have a surface-to-volume ratio comparatively greater than large ones, resulting in higher fiber content as fiber is mostly contained in the outmost layers of the kernel [32].

Total antioxidant capacity (TAC) was mostly affected by the year of growth and to a lesser extent by the entries and the preceding crop (Table 2). The year effect likely reflects the less favorable climatic conditions observed in 2022, characterized by higher average temperatures and less favorable rainfall timing during grain filling, than in 2023. This led to an increased biosynthesis of antioxidant compounds by the plant under abiotic stress conditions [33]. Both EPs showed a higher TAC than the variety Monnalisa, although only EP_Floriddia differed significantly (Table 2), suggesting a further role for these populations in the sector of healthy food besides sustainability and safeguarding agrobiodiversity.

### 3.3. Technological and Rheological Characterization

**Figure 2:**
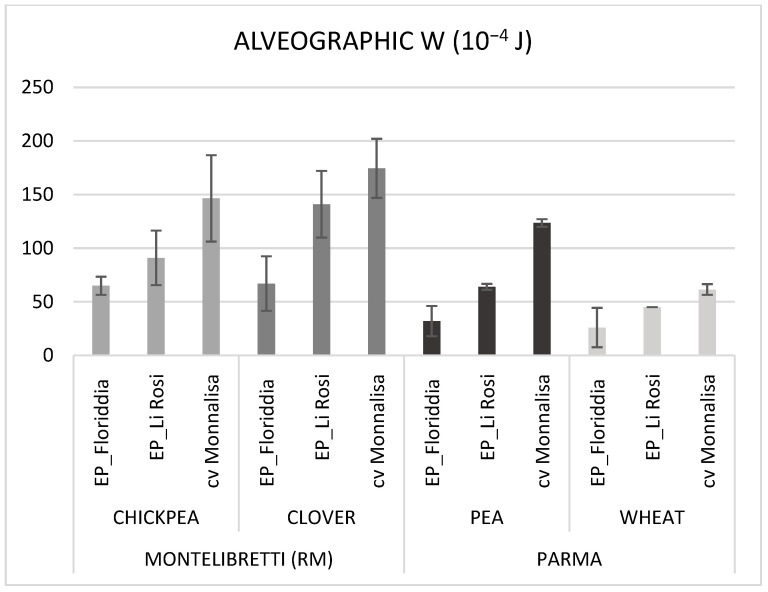
Mean alveographic W value of EP_Floriddia, EP-Li Rosi, and bread wheat cv Monnalisa over 2022 and 2023 growing seasons, in Montelibretti (RM) and Parma, after different preceding crops.

The SDS sedimentation volume is a test that provides information on the quantity and quality of proteins and on gluten characteristics [34]. The values are expressed in milliliters (mL) and increase as the quality of gluten proteins improves. Indeed, a higher sedimentation volume is generally associated with better quality of flour and a greater capacity to make products with the desired structure and texture [35,36]. Both EPs and the cultivar Monnalisa showed SDS volume ascribable to the n.1 quality class according to the UNI 10940:2001 [37] (≥40 mL), indicating good aptitude for bread-making (Table 3). This parameter was affected by location, years, and preceding crops. In particular, when the preceding crop was a legume, a significantly higher value of SDS was observed compared to wheat because of the higher protein content found in crops grown after legumes (Table 2). This was particularly true when the previous crop was clover. Despite the higher protein content and SDS values, gluten index (GI) assessment (Table 3) classified both evolutionary populations in the 3rd class of quality in UNI 10940:2001, falling in the range from 30 to 60% [37], with EP_Floriddia being the worst, whereas the cultivar Monnalisa showed a gluten index > 80% (1st class of quality). The low gluten quality of the two EPs, and especially of EP_Floriddia, was confirmed by their alveographic W, which was always <100 × 10^−4^ J (Table 3, Figure 2). Gluten strength, as revealed by alveographic W values, was significantly affected by entry, location, year, and preceding crop (Table 3). There was a significant entry × precessions interaction on W values, with clover precession scoring the highest W, whereas wheat precession determined a significant deterioration in dough strength (Table 3). These results support the observation that gluten quality is a trait distinct from gluten quantity, the latter being more closely related to the total protein content rather than to its quality. In fact, to more accurately assess gluten quality and consequently estimate the bread-making aptitude of a genotype, the gluten index and the W value are preferable to the SDS test as gluten strength predictors [35,36]. Indeed, the allelic composition of storage proteins by SDS-PAGE analysis of the two EPs revealed different protein patterns of high-molecular-weight glutenin subunits (HMW-GSs). In detail, in EP_Floriddia, the predominant HMW-GS compositions, according to the nomenclature of [38], were null, 1,2* (*Glu A1*); 6 + 8 (*Glu B1*); and 2 + 12 (*Glu D1*), whereas in EP_Li Rosi the predominant HMW-GS compositions were null, 1, 2* (*Glu A1*); 7, 18*, 20 (*Glu B1*); 5 + 12, 5 + 10, and 2 + 12 (*Glu D1*). The presence of HMW-GS 2 + 12 occurred in both EPs and the HMW-GS 20 in EP_Li Rosi, resulting in small-molecular-weight glutenin aggregates and low or scarce gluten quality [39]. However, EP_Li Rosi also presented the HMW_GS composition of 5 + 10, related to better baking aptitude [40].

Finally, all the entries showed dough with low tenacity (P) and high extensibility (L), as indicated by P/L values not exceeding 0.5 (Table 3). Though alveographic W was always <180, the P/L ratio results were quite balanced, suggesting that these populations could be suitable raw materials for the production of flat breads and other low-leavened baked products. Finally, the Elasticity Index (Ie) values, a key indicator of the functional quality of storage proteins associated with bread-making aptitude [41], exceeded 45% only in the cultivar Monnalisa (Table 3). In contrast, both EPs exhibited significantly lower values, particularly EP_Floriddia, thereby reinforcing the hypothesis that flours derived from evolutionary populations are more suitable for the production of flat breads. Also, [42], on the basis of rheological parameters, suggested that bread wheat EPs were indicated for brief-leavening bakery products.

The low gluten strength assessed by the GI and the alveographic parameters was confirmed by the farinographic test. A farinograph evaluates the behavior of flour during kneading. Long dough development time is a desirable feature for bread wheat flour, as well as a high water absorption capacity, high stability value, and low degree of softening [43]. These quality parameters are summarized by the farinograph quality number (IQ), which was affected by the location, the year, and the entry, with the EP_Li Rosi showing the best performance (Table 3), thanks mainly to the development time and stability of the dough. Notably, the IQ scores followed the same pattern as the other gluten quality assessments, specifically the SDS sedimentation test, gluten index, and alveographic W, which presented significantly higher values in 2022 than in 2023. Moreover, the worst performances were found when wheat was used as the previous crop. Environmental conditions such as mean temperature and precipitation during the grain-filling period can indeed influence rheological characteristics such as GI, alveographic, and farinographic parameters [44]. Temperature influences the duration of the grain-filling phase and the accumulation and polymerization of storage proteins: higher mean temperatures often accelerate maturation, shorten the grain-filling period, and can lead to a less optimal gluten network, even if total protein content increases. On the other hand, wet conditions during grain filling may dilute or degrade functional proteins and result in weaker gluten performance [45]. Nevertheless, it is noteworthy that while environmental factors affect the absolute values of rheological parameters, the relative ranking of the entries remained determined predominantly by genetic factors (such as HMW-GS alleles) rather than environment alone [44]. Therefore, in our study, EP-Floriddia showed the worst results in terms of gluten strength in both years and locations.

The falling number (FN) is used for assessing the baking quality of wheat flour in relation to the amylase activity. In the first growing season, FN was higher than 300 s (Table 3), indicating low amylase activity, which could determine a delay in fermentation and products with a hard crust and low bread volume [46]. Indeed, as observed by [47], a higher FN is associated with a higher air temperature during the grain filling. This aligns with the higher average temperature observed during grain filling in 2022, which likely inhibited α-amylase activity. The good bread-making aptitude of EP_Li Rosi was confirmed by a FN value very similar to cv Monnalisa, which indicated bread with fair crust texture and loaf volume. Notably, the two EPs differed significantly from each other for six of the seven rheological and technological characteristics, i.e., GI, W, P/L, Ie, IQ, and FN.

## 4. Conclusions

The results of this study indicate that wheat evolutionary populations (EPs) can successfully adapt to the Italian environment, expressing their nutritional and technological potential. Thus, they represent promising materials for enhancing diversification in nutrition, contributing to the development of healthier foods and promoting more sustainable agriculture.

The technological and rheological characteristics observed in the two EPs can be explained by their origin. Both populations were developed in Syria using parental material selected for North Africa and the Near East, where flat bread is the most common type of bread, especially in rural areas.

Given that EPs are heterogeneous plant materials derived from crossing hundreds of varieties, it is expected that they evolve differently in various locations as a result of natural selection. However, it is known that quality traits, unlike yield and disease resistance, are not directly influenced by natural selection [48,49]. Therefore, it was particularly interesting to find that the two EPs differed in six of the seven rheological and technological characteristics, as well as in protein content, 1000-kernel weight, and ash content. Since these traits are not directly associated with fitness, it could be argued that they are likely linked to other traits that are directly influenced by natural selection.

Technological analysis highlighted that, compared to wheat monocultures, legume preceding crops resulted in higher values of alveographic W, SDS sedimentation volume, and protein content in both evolutionary populations, even though they were characterized by weak gluten. Notably, “weak gluten” should not be interpreted as poor quality, but rather as an indication of suitability for specific processing sectors such as flat breads, biscuits, and specific pastry products. Among the two populations, EP_Li Rosi exhibited the best performance in terms of processability into baked products. The differences observed between various legumes preceding crops offer farmers an opportunity to manage and improve the quality of flour produced from EPs.

Moreover, nutritional analyses showed that both bread wheat EPs had higher protein content and total antioxidant capacity compared to the uniform variety Monnalisa, even with comparable grain yield.

Overall, the results obtained over these two years demonstrate good technological and nutritional performance for the evolutionary populations. The proposed strategy supports their feasibility as raw materials for organic cereal food production. Additionally, this study highlights the ability of EPs to adapt to different environments and withstand diverse climatic conditions.

These findings underline the potential of wheat evolutionary populations as valuable raw materials for developing nutritious, sustainable, and diversified cereal-based foods. Future research should further investigate the mechanisms linking adaptability and quality traits, as well as assess processing performance and consumer acceptance under different production contexts.

Legal authorization to market seeds of evolutionary populations is expected to enhance their availability, not only for small-scale farmers but also for cereal processors, who often seek diverse and locally adapted raw materials. This increased accessibility may foster the development of more resilient and regionally tailored organic value chains across the European Union.

## Figures and Tables

**Table 1:** Means and mean squares of physico-chemical characteristics of three bread wheat entries (two evolutionary populations and cv Monnalisa) grown for two cropping seasons in two locations following different preceding crops.

Main Factors	TKW (g)		TW (Kg/hL)	ASH (%)
**Year**				
2022	43.0 a		80.0 a	1.98 b
2023	43.6 a		75.4 b	2.03 a
**Location**				
Montelibretti (RM)	44.6 a		77.5 a	2.03 a
Parma	42.0 b		78.0 a	1.98 b
**Preceding Crop**				
Chickpea	46.1 a		78.2 ab	2.05 a
Clover	43.2 b		76.7 b	2.02 ab
Wheat	41.6 b		76.8 b	1.96 b
Pea	42.4 b		79.1 a	2.00 ab
**Entries**				
EP_Floriddia	46.6 a		76.7 a	2.22 a
EP_Li Rosi	41.4 b		77.8 a	1.88 b
cv Monnalisa	42.0 b		78.6 a	1.91 b
CV%	4.2		3.5	3.4
**Source of variation**	**d.f.**			
Years	1	5.6	258.5 ***	0.03 *
Locations	1	82.1 ***	2.7	0.03 *
Preceding crops	2	28.1	23.7	0.01 **
Entries	2	127.4 **	14.1	0.570
Entries × Precessions	6	3.0 ***	3.5	0.03 *
Entries × Years	2	10.9	10.1	0.01 **
Residual	32	4.0	7.3	0.005
Total	47	12.3	13.5	0.033

Means followed by the same letter (s) in a column for each variable are not significantly different based on Fisher’s L.S.D. at P = 0.05; TKW = thousand kernel weight; TW = test weight; ASH = ash content; CV = coefficient of variation; d.f. = degrees of freedom; significant at * = *p* < 0.05; ** = *p* < 0.01; *** = *p* < 0.001.

**Table 2:** Means and mean squares of nutritional characteristics of three bread wheat entries (two evolutionary populations and cv Monnalisa) grown for two cropping seasons in two locations following different preceding crops.

Main Factors		PC (%)	TS (%)	TDF (%)	TAC (mmol TEAC/kg)
**Year**					
2022		12.7 b	62.4 b	11.8 b	42.2 a
2023		14.4 a	71 a	13 a	35.7 b
**Location**					
Montelibretti (RM)		14.6 a	63.7 b	12.1 b	39.8 a
Parma		12.5 b	69.7 a	12.7 a	38.1 a
**Preceding Crop**					
Chickpea		13.9 b	63.2 b	12 b	38.4 ab
Clover		15.3 a	64.2 b	12.2 b	41.2 a
Wheat		11.7 c	71.9 a	12.6 ab	36.2 b
Pea		13.2 b	67.5 ab	12.9 a	40.0 a
**Entries**					
EP_Floriddia		14.9 a	64.9 a	12.6 a	41.1 a
EP_Li Rosi		13.6 b	68.3 a	12.5 a	39.1ab
cv Monnalisa		12.1 c	67.0 a	12.1 a	36.8 b
CV%		8.7	8	6.4	9.9
**Source of variation**	**d.f.**				
Years	1	34.8 ***	877.4 ***	19.00 ***	499.33 ***
Locations	1	53.8 ***	435.9 ***	5.48 **	33.99
Preceding crops	2	12.4 ***	60.5	0.52	66.7 *
Entries	2	31.4 ***	48.1	0.97	74.26 *
Entries × Precessions	6	1.8	33.6	1.40	7.48
Entries × Years	2	1.2	117.9 *	0.62	0.77
Residual	32	1.4	28.7	0.63	14.83
Total	47	5.0	61.9	1.22	28.45

Means followed by the same letter (s) in a column for each variable are not significantly different based on Fisher’s L.S.D. at P = 0.05; PC = protein content; TS = total starch; TDF = total dietary fiber; TAC = total antioxidant capacity; CV = coefficient of variation; d.f. = degrees of freedom; significant at * = *p* < 0.05; ** = *p* < 0.01; *** = *p* < 0.001.

**Table 3:** Mean and mean squares of rheological and technological characteristics of three bread wheat entries (two evolutionary populations and cv Monnalisa) grown for two cropping seasons in two locations following different preceding crops.

Main Factors		SDS (mL)	GI (%)	Alveograph Parameters			IQ	FN (s)
				W (10^−4^ J)	P/L	Ie (%)		
**Year**								
2022		50.1 a	63.9 a	96.7 a	0.41 a	38.0 a	35.3 a	362.3 a
2023		42.9 b	53 b	76.7 b	0.41 a	29.1 b	29.1 b	244.3 b
**Location**								
Montelibretti (RM)		51.7 a	59.6 a	114.5 a	0.36 b	39.2 a	35.9 a	268.4 b
Parma		41.3 b	57.4 a	58.9 b	0.46 a	27.9 b	28.5 b	338.3 a
**Preceding Crop**								
Chickpea		48 b	61.3 a	101.3 b	0.34 b	38.1 ab	37.8 a	255.3 c
Clover		55.4 a	57.9 ab	127.7 a	0.38 b	40.4 a	34.0 ab	281.5 bc
Wheat		37.4 c	65.5 a	44.4 d	0.52 a	24.7 c	26.0 c	325.8 ab
Pea		45.1 b	49.3 b	73.5 c	0.40 b	31.0 b	31.1 bc	350.8 a
**Entries**								
EP_Floriddia		44.5 a	29.1 c	47.8 c	0.48 a	21.1 c	28.9 b	344.9 a
EP_Li Rosi		47.6 a	52.4 b	85.5 b	0.36 b	31.1 b	38.1 a	283.6 b
cv Monnalisa		47.3 a	94.0 a	126.8 a	0.39 b	48.4 a	29.6 b	281.4 b
CV%		11.1	24.1	18.0	18.7	9.3	23.0	23.2
**Source of variation**	**d.f.**							
Years	1	623.5 ***	1419 **	4816 ***	0.008	950.5 ***	456 **	167,088 ***
Locations	1	1312.5 ***	59	37,085 ***	122.008 ***	1550.4 ***	645 **	58,520 **
Preceding crops	2	341.4 ***	825 *	4636 ***	46.208 **	134.2 ***	120	3929
Entries	2	47.4	17,314 ***	25,004.2 ***	59.908 ***	3061.1 ***	421 **	20,790 *
Entries × Precessions	6	37.7	233	1339 ***	13.375	42.8 *	55	2701
Entries × Years	2	51.7	756 *	14	236.058 ***	122.3 ***	141	13,739
Residual	32	26.7	198	243	5.868	13.5	55	4946
Total	47	82.9	1000	2565	23.285	209.0	100	10,151

Means followed by the same letter(s) in a column for each variable are not significantly different based on Fisher’s L.S.D. at P = 0.05; SDS = sedimentation volume; GI = gluten index; Ie = Elasticity Index; IQ = index of farinograph quality; FN = falling number; CV = coefficient of variation; d.f. = degrees of freedom; significant at * = *p* < 0.05; ** = *p* < 0.01; *** = *p* < 0.001.

## Data Availability

The original contributions presented in this study are included in the article. Further inquiries can be directed to the corresponding author.
